# Endothelial Function Is Associated with White Matter Microstructure and Executive Function in Older Adults

**DOI:** 10.3389/fnagi.2017.00255

**Published:** 2017-08-02

**Authors:** Nathan F. Johnson, Brian T. Gold, Christopher A. Brown, Emily F. Anggelis, Alison L. Bailey, Jody L. Clasey, David K. Powell

**Affiliations:** ^1^Department of Rehabilitation Sciences, Division of Physical Therapy, University of Kentucky Lexington, KY, United States; ^2^Department of Neuroscience, University of Kentucky Lexington, KY, United States; ^3^Magnetic Resonance Imaging and Spectroscopy Center, University of Kentucky Lexington, KY, United States; ^4^Sanders-Brown Center on Aging, University of Kentucky Lexington, KY, United States; ^5^Department of Medicine, University of Tennessee College of Medicine Chattanooga Chattanooga, TN, United States; ^6^Department of Kinesiology and Health Promotion, University of Kentucky Lexington, KY, United States; ^7^Clinical Services Core, University of Kentucky Lexington, KY, United States

**Keywords:** aging, endothelial function, reactive hyperemia, diffusion tensor imaging, executive function, white matter hyperintensity

## Abstract

Age-related declines in endothelial function can lead to cognitive decline. However, little is known about the relationships between endothelial function and specific neurocognitive functions. This study explored the relationship between measures of endothelial function (reactive hyperemia index; RHI), white matter (WM) health (fractional anisotropy, FA, and WM hyperintensity volume, WMH), and executive function (Trail Making Test (TMT); Trail B − Trail A). Participants were 36 older adults between the ages of 59 and 69 (mean age = 63.89 years, SD = 2.94). WMH volume showed no relationship with RHI or executive function. However, there was a positive relationship between RHI and FA in the genu and body of the corpus callosum. In addition, higher RHI and FA were each associated with better executive task performance. Tractography was used to localize the WM tracts associated with RHI to specific portions of cortex. Results indicated that the RHI-FA relationship observed in the corpus callosum primarily involved tracts interconnecting frontal regions, including the superior frontal gyrus (SFG) and frontopolar cortex, linked with executive function. These findings suggest that superior endothelial function may help to attenuate age-related declines in WM microstructure in portions of the corpus callosum that interconnect prefrontal brain regions involved in executive function.

## Introduction

Age-related decreases in vascular health are a common finding in the literature (Brown and Thore, 2011) and represent one of many potential mechanisms that contribute to declines in the integrity of the aged brain (Duncan, 2011). Identifying clinical markers of vascular health that serve as surrogate signs of brain health is paramount for early intervention and prevention efforts. Ideal markers of vascular health would be non-invasive, able to detect early changes in vascular function, easily administered in clinical settings, and related to neuroimaging techniques that are sensitive to age-related vascular decline.

Neuroimaging indicators of white matter (WM) health, including fractional anisotropy (FA) and WM hyperintensities (WMHs), are sensitive biomarkers of age-related vascular decline (Rowe Bijanki et al., 2013; Singer et al., 2014). WMHs are associated with increased pulse-wave velocity, a measure of conduit artery stiffness (Singer et al., 2014), and FA is significantly decreased in vascular disease (Rowe Bijanki et al., 2013). In addition, changes in FA appear to precede the manifestation of irreversible WM lesions (de Groot et al., 2013; Pelletier et al., 2015), and are predictive of future cerebrovascular incidents (Evans et al., 2016). Despite this evidence, less is known about the relationship between these neuroimaging predictors and early detectors of cardiovascular disease, such as endothelial function (Bruno et al., 2014).

The vascular endothelium is a single cell layer lining all blood vessels. It plays a critical role in regulating vascular tone by mediating the relationship between luminal blood flow and arterial smooth muscle. When compromised, the endothelium contributes to the pathogenesis of vascular disease (Cahill and Redmond, 2016). Advancing age is associated with endothelial dysfunction (Seals et al., 2014), and endothelial dysfunction is associated with Alzheimer’s disease and vascular dementia (Dede et al., 2007; Zuliani et al., 2008). Moreover, blood markers of chronic endothelial dysfunction (i.e., thrombomodulin and tissue factor) are associated with rarefaction of WM (Hassan et al., 2003). Collectively, these findings suggest that endothelial function may play a critical role in combating age-related declines in brain health.

Endothelial function can be measured non-invasively through the use of digital pulse amplitude technology, which allows for the assessment of vascular function at the fingertip. A fingertip plethysmograph capable of sensing volume changes at the digit is used to measure arterial pulsation at rest, and following occlusion induced reactive hyperemia (Axtell et al., 2010). This measure of peripheral arterial tone (PAT) is correlated with changes in vascular tone using flow-mediated dilation techniques (Kuvin et al., 2003), and has been shown to improve following healthy lifestyle modifications (Fisher and Hollenberg, 2006; Barringer et al., 2011).

Little is known about the relationship between endothelial function and WM health. Endothelial cells mediate vessel caliber (Gori et al., 2016), and age-related endothelial dysfunction may induce vasoconstriction and chronic hypoperfusion of WM (Pantoni, 2002; Seals et al., 2014). Ischemia can then lead to myelin degeneration and selective oligodendrocyte death (Pantoni et al., 1996; Petito et al., 1998). Recent findings support this mechanism by demonstrating a relationship between microvessel caliber and normal appearing WM. For example, Mutlu et al. (2016) found that narrower retinal arterioles, surrogate markers of cerebrovascular health (Ikram et al., 2006; de Jong et al., 2011), were associated with poorer WM microstructure (Mutlu et al., 2016). Despite this evidence, a greater understanding of this potential relationship is important because maintenance of WM health is required for proper transmission of information between cortical regions. Furthermore, age-related changes in WM health are associated with alteration in functional brain response and poorer cognitive performance on executive tasks (Hakun et al., 2015b; Zhu et al., 2015) and executive function (Charlton et al., 2006; Gold et al., 2010b).

The Trail Making Test (TMT) is a reliable and valid assessment of executive function that is related to WM health and overall brain health (Reitan, 1958; Kinnunen et al., 2011). Little is known about the relationship between executive function and endothelial health in older adults without cardiovascular disease. Tsao et al. (2013) reported a positive relationship between brachial artery diameter, flow velocity, and logical memory, but did not observe a relationship between reactive hyperemia and executive function. However, Lim et al. (2015) recently reported a positive relationship between reactive hyperemia and executive function. Of note, executive function is sensitive to modifiable lifestyle variables (i.e., exercise) that can impact vascular health (Colcombe and Kramer, 2003).

In the present study, we used a non-invasive measure of PAT to test the hypothesis that endothelial function is associated with WM health and executive function. We first explored potential relationships between WM health and reactive hyperemia using an unbiased voxelwise approach (FA), and objective quantification of WM lesions (WMHs). We then expanded on these findings by exploring the potential relationships between a measure of executive function, the TMT, and both WM health and reactive hyperemia. Finally, we used tractography methods to determine the anatomical connectivity patterns of WM tract clusters showing a correlation with endothelial function in the voxelwise results.

## Materials and Methods

Forty-two community dwelling healthy volunteers (14 males) participated in this study (mean age = 63.89 years, SD = 2.94). Participants provided written informed consent in a manner approved by the University of Kentucky Institutional Review Board and were monetarily rewarded for participating. All subjects gave written informed consent in accordance with the Declaration of Helsinki. The protocol was approved by the University of Kentucky Institutional Review Board. Six of the forty-two participants were excluded from the study. Of these six participants one reported a diagnosis of Reynaud’s disease, three failed to complete the magnetic resonance imaging (MRI) portion of the study, and two opted to terminate the test of endothelial health due to discomfort. The 36 remaining participants (13 males) ranged in age from 59 to 69 (mean age = 63.7 years, SD = 2.9). Two of the remaining 36 participants did not complete the TMT (described below) due to time limitations, and two were eliminated due to TMT outlier status (>2.5 SD above the mean). Participants met all criteria for participating in a MRI study. Exclusion for this study included history of a major head injury and/or concussion, neurological disorder (e.g., stroke, seizure), reported psychotropic drug use, or the presence of metal fragments and/or metallic implants that could cause bodily injury or disrupt the magnetic field. This information was verified during phone interviews with each participant. Although we did not screen for current or past alcohol consumption, no participant reported consuming greater than four drinks per week.

### Trail Making Test (A and B)

Executive function was evaluated using the TMT. The TMT is a reliable assessment of executive function that is related to WM health and overall brain health (Reitan, 1958; Kinnunen et al., 2011). Trails A and B each contain 25 circles distributed over a single sheet of paper. The circles in Trail A are numbered 1 through 25 and participants are instructed to draw lines to connect the numbers in ascending order. The circles in Trail B are labeled with either numbers, 1 through 13, or letters, A through L. Participants are instructed to connect the circles in an ascending pattern while alternating between numbers and letters (i.e., 1-A-2-B-3, etc.). The difference in the time it takes to complete Trail A and B (Trail B − Trail A) is used as a measure executive function.

### Endothelial Peripheral Arterial Tone (EndoPAT)

Reactive hyperemia was evaluated using the EndoPAT 2000 (Itamar Medical, Israel). PAT is a measure of the pulsatile volume changes to a reactive hyperemia challenge at the fingertip. It was measured using proprietary non-invasive finger PAT probes. The reactive hyperemia procedure consisted of a 10-min baseline recording with the participant in a relaxed seated position with both arms resting on grooved arm rests. A blood pressure cuff was used to occlude blood flow to the non-dominant arm for 5-min. Resting systolic blood pressure was used to determine the appropriate level of cuff inflation. Finally, post-occlusion pulsatile volume changes were recorded for 5-min. The ratio between the post- to pre-occlusion average signal size was then calculated to determine each participant’s reactive hyperemia index (RHI).

### High-Resolution Anatomical Image

Data were acquired on a 3T TIM Siemens scanner at the University of Kentucky’s Magnetic Resonance Imaging and Spectroscopy Center. A 32-channel head coil was used. A single high-resolution, 3D anatomic image was acquired using a magnetization-prepared rapid gradient-echo (MPRAGE) sequence with the following parameters: echo time (TE) 2.26 ms, repetition time (TR) 2530 ms, field of view (FOV) of 256 mm, flip angle (FA) of 7°, and voxel size of 1 mm × 1 mm × 1 mm.

### Diffusion Tensor Imaging

Whole brain diffusion tensor images were acquired with 64 non-collinear encoding directions (*b* = 1000 s/mm^2^) and six images without diffusion weighting (*b* = 0 s/mm^2^, b0) using a double-spin echo EPI sequence (TR = 8000 ms, TE = 96 ms, FOV = 224 mm, 52 slices, 2 mm isotropic resolution).

### FLAIR Imaging for WMH

Fluid-attenuated inversion recovery (FLAIR) images were acquired from older adults with a fat saturated turbo-spin echo (TSE) sequence (TR = 9000 ms, TE = 89 ms, TI = 2500 ms, FA = 130°, acquisition matrix = 256 × 174 × 34, 1 mm × 1 mm × 4 mm voxels).

### Diffusion Tensor Imaging Processing and Analysis

All diffusion tensor imaging (DTI) data were processed and analyzed using the Functional MRI of the Brain (FMRIB) Software Library (FSL v4.1.5). Raw images were corrected for motion and residual eddy current distortion using a 12-parameter affine alignment to the corresponding b0 image via FSL’s Linear Image Registration Tool (FLIRT^1^). To exclude non-brain voxels, FMRIB’s brain extraction tool (BET v2.1) was used to generate brain masks (Smith et al., 2006). Tensor fitting and FA calculations were performed using FMRIB’s Diffusion Toolbox (FDT v2.0).

FSL’s Tract-Based Spatial Statistics (TBSS v1.2; (Smith et al., 2006,^2^) was used to register the FA images into MNI152 space and perform all subsequent voxel-wise analyses, as described in detail in our previous work (Gold et al., 2010a; Johnson et al., 2012). Briefly, these steps included removing likely outliers from the fitted tensor, non-linearly aligning all FA images to a target image, and resampling images to a 1 × 1 × 1 mm MNI152 space. Next, all MNI-transformed FA images were averaged to generate a mean FA image. This mean FA image was then used to create a common WM tract skeleton that was thresholded at a FA value of 0.2. Individual FA images were subsequently projected onto the FA skeleton in order to account for residual misalignments.

Multiple regression analysis was performed to explore potential relationships between reactive hyperemia and FA. Covariates of no interest, age and sex, were included in all analyses. FSL’s threshold-free cluster enhancement (TFCE) method was used to avoid the use of an arbitrary threshold in the initial cluster formation. A voxelwise permutation nonparametric test (using 5000 permutations) was employed. A threshold of *P* < 0.05 (corrected for multiple comparisons) was used to identify significant clusters. FSL’s tbss_fill function was used for visualization purposes.

### Diffusion Tensor Imaging Probabilistic Tractography

Probabilistic tractography was performed to determine the anatomical connectivity patterns of the cluster that was correlated with reactive hyperemia in the voxelwise results. FSL’s Bayesian Estimation of Diffusion Parameters Obtained using Sampling Techniques (BEDPOSTX) and probabilistic tracking (PROBTRACKX) tools (Behrens et al., 2003a) were used to perform the tractography. Both are part of FMRIB’s Diffusion Toolbox (FDT v2.0^3^).

The cluster identified in the voxelwise results corresponded to anterior portions of the corpus callosum, including the body and genu. Target masks were created in MNI space to determine the connectivity strengths of voxels within the corpus callosum cluster to different regions of prefrontal and sensorimotor cortices. Frontal pole (FP), middle frontal gyrus (MFG), superior frontal gyrus (SFG), inferior frontal gyrus (IFG), precentral gyrus (PrCG), and postcentral gyrus (PoCG) masks were created using the Harvard-Oxford Cortical Structural Atlas provided by FSL’s software package. Separate MFG, SFG and premotor (PrM) cortex masks were created as described in our previous work (Johnson et al., 2012). Next, MNI space to native diffusion space matrices were used to explore the connection strengths of subject’s native space voxels to different structural target masks in MNI space. Specifically, FLIRT was used to generate transformation matrices, and their inverses, between subject’s native diffusion space and T1 images, and between T1 images and MNI space. This was achieved using the FMRIB’s suggested parameters^4^. The tractography analysis was conducted using FSL’s ProbtrackX software (Behrens et al., 2003b, 2007), as described in detail in our previous work (Johnson et al., 2012; Hakun et al., 2015a).

A hard segmentation was performed between the seed cluster identified in the DTI voxelwise analysis and the seven cortical target masks^5^. Voxels corresponding to different target regions were isolated using FSL’s “fslmaths” utility, resulting in seven different seed cluster image volumes. FSL’s “fslstats” utility was then used to record the number of voxels within the seed cluster that corresponded to the respective target mask. The numbers of voxels were then normalized to the corresponding target mask [(number of voxels in seed cluster/number of voxels in the corresponding target mask) × 100] in order to control for differences in target mask size (i.e., cortical region size).

Analysis of variance (ANOVA) was used to compare the normalized number of voxels that were connected to the seven cortical masks. Bonferroni *post hoc* analyses were performed when significant differences were observed.

### FLAIR Imaging Analysis for WMH Assessment

WMH volumes were computed in older adults using an overall framework employed in our recent work (Smith et al., 2016). The series of steps included field correction using the N3 algorithm, T1 image averaging, skull-stripping and segmenting using Freesurfer. Subsequent manual editing was performed to remove artifacts particularly in regions between the lateral ventricles, at the base of the brain and at the level of the superior sagittal sinus. The skull-stripped T1 image was then registered to the FLAIR image using FLIRT. A WM mask was generated from the segmentation results from Freesurfer by combining left cortical WM, right cortical WM and WM hypo-intensities. This WM mask was registered to the FLAIR image using the same affine transformation generated above.

The WM mask was applied to the FLAIR image, and mean and standard deviation of WM signal intensities were estimated based on voxel histogram fitting using a two-gaussian model. The voxel intensity histogram was thresholded at a standard deviation of 2.33 from the mean of the dominant (normal appearing WM) gaussian fit. The volume of hyperintensities exceeding the threshold was recorded for each participant as whole-brain (total) WMH volume. In addition, a separate measure of frontal WMH volume was computed due to the importance of the frontal lobes to executive function. The frontal WMH measure was computed by applying the MNI Structural Atlas Frontal Lobe Mask, which was edited to include WM. Participants’ WMH volumes were corrected for their total intracranial volume (ICV).

## Results

Demographic, endothelial and executive function data are shown in Table 1. There was a significant difference in height and weight between genders in our study. Males participants were taller (*F*_(1,34)_ = 34.3, *p* < 0.0001) and weighed more (*F*_(1,34)_ = 8.8, *p* = 0.005). No sex difference was observed for RHI or executive function.

**Table 1:** Demographic data, reactive hyperemia index (RHI) and executive function.

Subjects	Age	Height (m)	Weight (kg)	RHI	Trail B − Trail A^§^ (s)
*n* = 36	63.8 (2.9)	1.68 (0.10)	73.4 (13.4)	1.73 (0.5)	35.4 (15.5)
Female *n* = 23	63.9 (2.8)	1.63 (0.07)	68.8 (10.9)	1.74 (0.4)	32.7 (14.7)
Male *n* = 13	63.3 (3.2)	1.78** (0.09)	81.3** (14.1)	1.72 (0.5)	41.4 (16.4)

Abbreviations: m, meters; kg, kilograms; s, seconds. ^§^Denotes n of 32. Note: values are means and values in parentheses are SD **P < 0.005.

**Figure 1:**
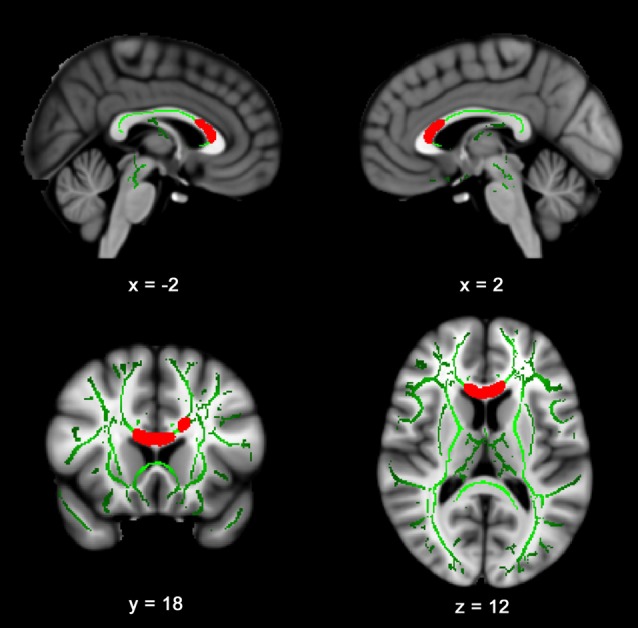
Reactive hyperemia index (RHI) is positively correlated with fractional anisotropy (FA) in the corpus callosum. Slices highlight the positive correlation observed in the genu and body of the corpus callosum after controlling for age and sex. The anatomic underlay used for illustration is the MNI152 T1-weighted 1 mm brain. The registered average FA skeleton is represented in green. The numbers below each slice represent the respective *x*, *y* and *z* coordinates of in MNI space.

**Figure 2:**
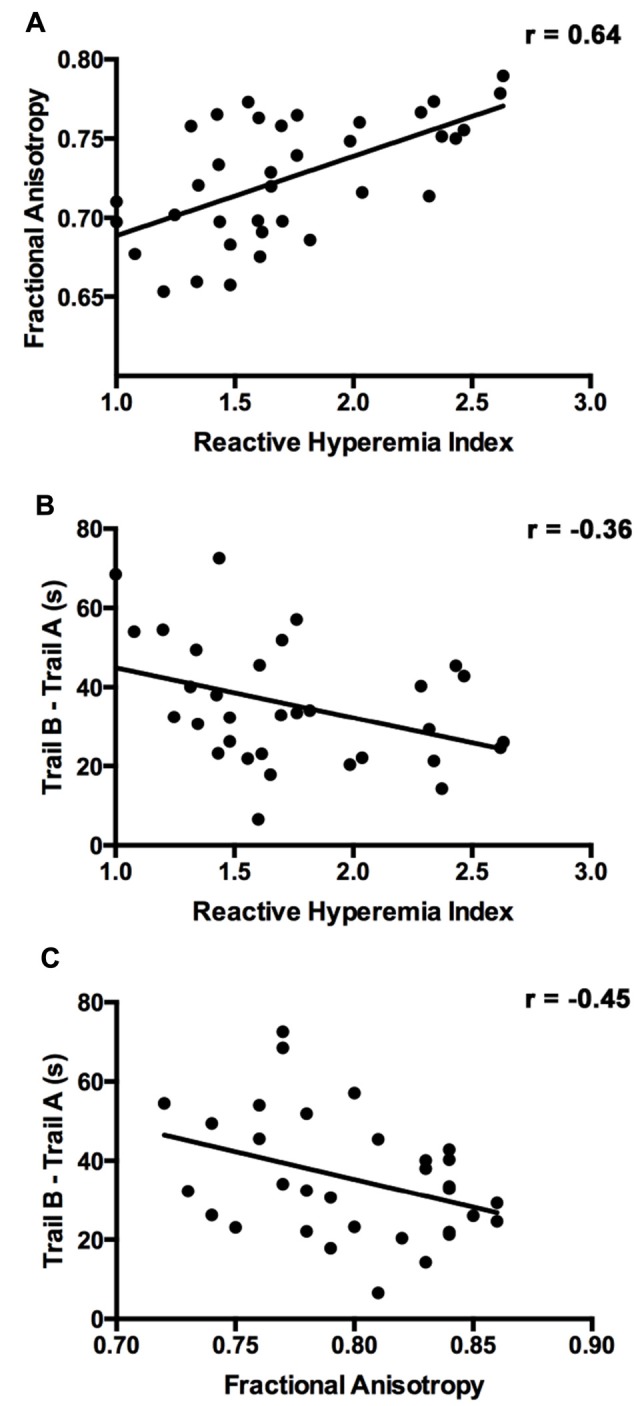
The relationship between RHI, FA, and executive function. **(A)** Scatter plot showing the relationship between RHI score and FA in the corpus callosum cluster identified in the diffusion tensor imaging (DTI) voxelwise analysis. **(B)** Scatter plot showing the relationship between executive function (Trail B − Trail A) and RHI. **(C)** Scatter plot showing the relationship between executive function (Trail B − Trail A) and FA.

Figure 1 presents the results of the voxelwise multiple regression analysis between reactive hyperemia and FA. A positive correlation was observed between RHI and FA in anterior portions of the corpus callosum including the genu and body. Subsequent statistical analyses revealed that FA (*r* = −0.445, *p* < 0.05) and RHI (*r* = −0.359, *p* = 0.05) were negatively associated with Trail B − Trail A performance. Scatter plots illustrating these relationships are presented in Figure 2.

No relationship was observed between frontal WMH volume and FA (*r* = 0.144, *p* = 0.44), reactive hyperemia (*r* = 0.274, *p* = 0.17), or executive function (*r* = 0.018, *p* = 0.93) when controlling for age and sex. In addition, no relationship was observed between global WMH volume and FA (*r* = 0.040, *p* = 0.83), reactive hyperemia (*r* = 0.095, *p* = 0.61), or executive function (*r* = 0.17, *p* = 0.40). Mean global WMH volume (% of ICV) was 0.30 (SD = 0.19), and mean frontal WMH volume was 0.12 (SD = 0.10).

**Figure 3:**
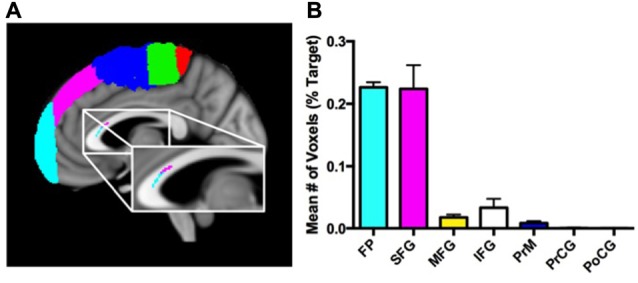
Connectivity patterns of corpus callosum voxels with cortical target masks. **(A)** Results of the hard segmentation from a sagittal slice of a single representative subject using seven target masks [frontal pole (FP) = cyan, inferior frontal gyrus (IFG) = gray, middle frontal gyrus (MFG) = yellow, superior frontal gyrus (SFG) = magenta, premotor cortex (PrM) = blue, precentral gyrus (PrCG) = green, postcentral gyrus (PoCG) = red)]. The anatomic underlay used for illustration is the MNI152 T1-weighted 1 mm brain. **(B)** Mean normalized number of voxels (expressed as a percentage of the total number of voxels in the target mask) connected to each of the seven different cortical masks.

Figure 3A presents the results from the hard segmentation of the seed cluster in a single, representative subject. The topography observed is consistent with that reported using comprehensive fiber tractography in the corpus callosum (Hofer and Frahm, 2006). Figure 3B presents a histogram plot of the normalized number of voxels within the significant corpus callosum cluster that were connected to each cortical mask. A significant effect for target mask was observed, *F*_(6,30)_ = 266, *P* < 0.0001. *Post hoc* comparisons using Bonferroni correction indicated that the normalized percentage of voxels corresponding to the SFG (*M* = 1.71, SD = 0.75) and frontopolar cortex (*M* = 0.99, SD = 0.14) were significantly greater than the normalized percentage of voxels corresponding to the PrM cortex (*M* = 0.47, SD = 0.22), IFG (*M* = 0.13, SD = 0.28), MFG (*M* = 0.27, SD = 0.30), PrCG (*M* = 0.04, SD = 0.06) and PoCG (*M* = 0.01, SD = 0.02; all *P*s < 0.0005).

## Discussion

The present study represents the first exploration of the relationship between endothelial function and brain structure. Our results build upon findings that vascular health may help to maintain the structural and functional integrity of the aged brain (Kennedy and Raz, 2009; Leritz et al., 2011; Gauthier et al., 2015). Specifically, we found that reactive hyperemia was associated with WM microstructure (FA) in anterior portions of the corpus callosum. Tractography results indicated that the region of the corpus callosum showing a significant relationship with an index of reactive hyperemia (RHI) contained fibers interconnecting a network of homologous prefrontal cortex regions, most prominently involving those linked with executive function. Moreover, both WM microstructure and RHI were related to a measure of executive function, the TMT. Unlike WM microstructure, frontal WMH volume was not related to reactive hyperemia or executive function. The implications of these findings are discussed below.

Advancing age is associated with deleterious changes in brain structure and cognition. Cumulative evidence suggests that age-related changes in vascular function contribute to these declines (Henry Feugeas et al., 2005; Giannakopoulos et al., 2007; Barnes, 2015). We therefore chose to focus the attention of this study on determining the relationship between endothelial function and neuroimaging markers of WM health. We observed a positive correlation between RHI and FA across the genu and anterior portions of the body of the corpus callosum, but observed no relationship between WMH volume and RHI. Our results build on previous findings that cardiorespiratory fitness, a benefactor of endothelial health, is related to WM microstructure (Marks et al., 2011; Johnson et al., 2012; Voss et al., 2013), and support findings that WM microstructure may be a more sensitive, and early, marker of the insidious decline of WM health (Pelletier et al., 2015).

The pathogenesis of WM damage as a result of age-related changes in vascular health is unknown. It has been proposed that small vessel disease leads to an initial loss of smooth muscle, followed by vessel wall thickening, reduced cerebral blood flow, chronic ischemia, demyelination, and subsequent leukoaraiosis (Greenberg et al., 2009; Pantoni, 2010). A recent review by Poggesi et al. (2016) offers insight into the potential pathophysiology behind our findings. Specifically, the response of endothelial cells to pathologic stimuli promote the initiation of vascular compromise (Virmani et al., 2000), whereas the presence of WMHs are associated with more chronic phases of vascular compromise, including breakdown of the blood-brain barrier (Young et al., 2008). More specifically, systemic indicators of inflammation, such as endothelial expression of C-reactive protein (CRP), are negatively correlated with FA, despite showing no relationship with WMHs (Wersching et al., 2010; Miralbell et al., 2012). WMHs, on the other hand, are strongly associated with pulse-wave velocity (Singer et al., 2014), a measure indicative of chronic vascular compromise, arterial stiffness. Thus, RHI appears to be a marker of endothelial-dependent microvascular function, which has also been reported in high-risk cardiovascular patients (Matsuzawa et al., 2010; Michelsen et al., 2016). Our findings suggest that declines in endothelial function may represent an early, preclinical indicator of WM health.

Tractography results suggest that the potential benefits of endothelial health are associated with a functionally heterogenous portion of the corpus callosum. For example, endothelial health was positively associated with WM anatomical connections between homologous prefrontal regions. In particular, the number of corpus callosum seed voxels interconnecting the FP and SFG were significantly higher than those interconnecting homologous somatosensory or pre-motor cortices. The FP and SFG contribute to a range of high-level cognitive functions including relational integration (Bunge et al., 2009), subgoal monitoring and integration during working memory (Braver and Bongiolatti, 2002), task switching (Armbruster et al., 2012) and inhibitory control (Li et al., 2006).

The location of the relationship between endothelial function and WM microstructure may be particularly important to the study of aging given that these tracts are especially vulnerable to age-related declines (Sullivan and Pfefferbaum, 2006; Bennett et al., 2010). The corpus callosum is one of the brain’s main commissural tracts that permits interhemispheric transmission of sensory, motor and cognitive information, and we have previously shown that WM microstructure in the genu of the corpus callosum mediates the relationship between age and the blood oxygen level dependent (BOLD) signal during task switching (Zhu et al., 2014). Collectively, these findings suggest that endothelial function is related to WM tracts important for maintaining neural efficiency and executive function.

To determine the functional consequences associated with higher WM microstructure and superior endothelial function, we explored the relationship between a measure of executive function, the TMT, and both FA and RHI. Slowed performance on Trail B compared to Trail A (Trail B − Trail A) represents an impaired ability to execute and modify a planned action to switch between sequential numbers and letters. Thus, smaller differences in Trail B compared to Trail A suggest superior executive function. We observed that higher FA in the genu and body of the corpus callosum, and higher RHI values, were associated with smaller differences in Trail B compared to Trail A. These findings support previously reported associations between reactive hyperemia and executive function (Lim et al., 2015). Thus, superior endothelial function may serve as a peripheral marker of WM and cognitive health.

The present study has several caveats that highlight areas that need further investigation. First, the cross-sectional nature of our study limits the ability to draw causal conclusions about endothelial function and WM microstructure. The relationship observed in the present study serves to justify future intervention studies to determine if improved endothelial health boosts WM microstructure. Second, longitudinal designs are required to determine if temporal changes in endothelial health and WM microstructure track with age-related declines in executive function. Third, this study focused on peripheral measures of endothelial function by measuring reactive hyperemia at the fingertip. It is important to note that cerebral vessels are functionally and structurally different from systemic vessels (Cipolla, 2009), requiring future studies to explore more central measures of flow-mediated dilation. Finally, the presence of cerebral microbleeds may also play a critical role in WM health. Future studies should acquire susceptibility-weighted images to determine the relationship between reactive hyperemia, WM microstructure and degree of microbleeds.

In conclusion, our results demonstrate that a peripheral measure of endothelial function, RHI, is positively correlated with WM microstructure in the genu and body of the corpus callosum in older adults, but is not related to WMH volume. The observed RHI-WM relationship was observed in the genu of the corpus callosum, a region sensitive to age-related declines. The results from tractography analyses suggest that portions of the corpus callosum most strongly correlated with WM microstructure were those interconnecting homologous prefrontal cortex regions involved in higher-level cognitive processes. These findings motivate future longitudinal studies aimed to determine if increasing endothelial function, through lifestyle modification, attenuates age-related declines in WM microstructure and executive function.

## Author Contributions

NFJ: Co-I on grant funding the project. NFJ is the corresponding author and was involved in all data collection, analysis and interpretation. BTG: substantial contributions to the acquisition, analysis and interpretation of all data; critical revision of the submitted manuscript. CAB: developing and conducting white matter hyperintensity analysis, data interpretation, contributor to content of manuscript. EFA: conducting white matter hyperintensity analysis, contributor to content of manuscript. ALB and JLC: reactive hyperemia data collection, data interpretation, contributor to content of manuscript. DKP: Co-I on grant funding the project. DKP was involved with all data collection, analysis and interpretation.

## Conflict of Interest Statement

The authors declare that the research was conducted in the absence of any commercial or financial relationships that could be construed as a potential conflict of interest.

## Footnotes

^1^https://fsl.fmrib.ox.ac.uk/fsl/fslwiki/FLIRT

^2^http://www.fmrib.ox.ac.uk/fsl/tbss/

^3^https://fsl.fmrib.ox.ac.uk/fsl/fslwiki/FDT

^4^https://fsl.fmrib.ox.ac.uk/fsl/fslwiki/FDT/UserGuide#Registration_within_FDT

^5^https://fsl.fmrib.ox.ac.uk/fsl/fslwiki/FDT/UserGuide#find_the_biggest
